# Using the Age-Friendly Health Systems Framework to Track Wellness and Health Promotion Priorities of Older Adults in the Global Community

**DOI:** 10.3390/ijerph20054617

**Published:** 2023-03-06

**Authors:** Nina Tumosa

**Affiliations:** Senior Public Health Advisor–Geriatrics, Division of Medicine and Dentistry, Health Resources and Services Administration (HRSA), U.S. Department of Health and Human Services (HHS), Rockville, MD 20857, USA; ntumosa@hrsa.gov

The promotion of health and wellness interventions for older adults is important in controlling the onset and progression of disabilities as well as disease in these individuals. Well-established health promotion strategies used by healthcare providers to promote health and wellness in older adults have included primary prevention, such as health education, secondary prevention, such as screenings for geriatric syndromes, and tertiary prevention, such as managing chronic conditions to prevent disability [1], as well as behavior modifications and health communication [2].

The arrival of the COVID-19 pandemic greatly affected wellness and health promotion education delivery strategies, primarily with the expansion of the use of telehealth, including telemonitoring, telementoring, teleprecepting, and teleconferencing, and synchronous as well as asynchronous webinars to promote the health education of both providers and older adults worldwide [3]. 

The rapid global spread of COVID-19 and the adoption of wide-spread quarantines created a new world of public health protocols and strategies in which the health of the community was, at least temporarily, placed ahead of the health of individuals. Thus, the COVID-19 pandemic has also impacted screening priorities and the management of chronic conditions in patients with COVID-19 [4] and long COVID [5] who already have hypertension, diabetes mellitus, chronic obstructive pulmonary disease, or chronic kidney disease [4,5], as well as dementia and psychiatric disorders [5]. Given these difficult and rapidly changing circumstances, are there mechanisms in place that will allow for the creation of didactic and experiential clinical paradigmatic shifts to promote healthy ageing? Thankfully, the answer is yes.

The World Health Organization (WHO) is an active proponent for creating age-friendly communities that promote healthy ageing by addressing eight domains of livability [6]. Each of these eight domains provides new opportunities for innovations in promoting health and wellness. Health services and support (one of the eight domains) encourage the development of new ways to promote health and wellness for older adults through new and improved educational, clinical, and public health programming. An innovative development has been the introduction of the concept of an Age-Friendly Health Systems (AFHS) framework [7], which uses the 4Ms concept to identify and address health concerns and priorities. The 4Ms are (1) what matters to an older adult (i.e., ask older adults what their health and wellness goals are), (2) medication, (3) mobility, and (4) mentation [3], and they provide educators, clinicians, and administrators with an age-friendly framework within which to provide care. The use of the 4Ms has propelled the age-friendly movement beyond cities and communities and into ecosystems that include lived environments, healthcare systems, and public health systems [8], just in time to allow a robust response to COVID-19. 

A question worth asking is whether the 4Ms can be used to support a rapid response to a newly emerging disease such as COVID-19, which has unfortunately launched an atmosphere of distrust and fear on the parts of both providers and patients. Can a common language of geriatric care and goals help people understand geriatrics and the need for quick changes to promote wellness and health in vulnerable older adults when the entire health system is under siege? Fortunately, the recent introduction of the AFHS framework, through which geriatric care is taught and delivered, was well underway when the COVID pandemic arrived [9] and provides us with a satisfactory answer. 

This editorial reviews how the innovative projects introduced in this Special Issue represent the current state of age-friendly healthcare on four continents—Europe, Asia, Australia, and North America. The articles in this Special Issue provide an updated view of how current geriatric research and clinical care projects are addressing the health and well-being of older adults. 

This Special Issue covers both old and new topics, indicating that while we are still working to address some old priorities, new ones are emerging as demographic changes and the COVID-19 pandemic impact global health. The Special Issue highlights several approaches to healthy ageing that should prove to be helpful to healthcare providers, direct care workers, students, faculty, patients and their families, and caregivers on how to identify evidence-based practices that support the well-being of the older adults for whom they provide care.

The articles published in this Special Issue represent a convenience sample of peer-reviewed articles, submitted to a single journal, between March 2021 and February 2023. These published papers have been reviewed to determine which of the 4Ms of the age-friendly concepts were addressed. This editorial discusses the 4Ms topics that were presented in each paper and how this reflects the status of wellness and health promotion for older adults.

The topics covered by each article were assigned to one of the 4Ms in Table 1.

Wellness and health promotion for older adults is a global task. Tracking what the current priorities are in wellness and health promotion can be simplified by the application of the 4Ms of the AFHS framework to the overall structure of what is currently receiving attention from scholars, researchers, and policymakers. 

The topic of what matters to older adults was addressed in many of the papers in this Special Issue. 

Many older adults are concerned about food insecurity, the desire for autonomy, the desire to be respected for who they are and what they have accomplished, and the respect for their wishes for specific end-of-life care. New topics of concern, including a heightened recognition of the value of social interactions, the need for access to a trained healthcare workforce, the need to maintain personal resilience, and the increased value of telemedicine to access both education and clinical care, have been impacted by the COVID pandemic.

The topic of mentation also shows both old and new priorities. Old priorities include the screening, diagnosis, management, and treatment of cognitive impairments, as well as being able to properly differentiate between dementia, delirium, and depression. New priorities include an emphasis on the need to address anxiety, loneliness, resiliency or well-being, stress, and social isolation, all important components of mentation. Communication among team members, including patients, becomes even more important when addressing these new topics in mental health, which still retain the aura of stigma [36]. 

Some of the projects represented within this Special Issue address new topics of interest, while some do not. The topics of interest for both medication and mobility do not appear to have changed much in response to the pandemic, whereas the topics of concern for what matters and mentation have been affected by the pandemic. 

Medication concerns for older adults have long revolved around polypharmacy, medication reconciliation, and screening for common diseases or syndromes for which new medications are constantly being created. Prior to the COVID pandemic, infectious disease discussions and decision making regarding vaccinations were long-standing topics of interest; however, none of the papers in this Special Issue addressed issues related to access, workforce training on vaccine options, administration, delivery, storage, safety, and side effects, as well as the effect of disparities on health outcomes. 

The need to maintain mobility and strength through exercise remains a constant struggle for ageing older adults, and current research efforts appear to continue to concentrate on exercise plans, fall prevention, and fall screening. Only one paper addresses how to predict fall risk [28]. These results suggest that, during a pandemic, falls remain a concern but not necessarily a priority.

However, these current priorities can quickly change. Hypothetically, if a similar analysis were performed on 24 new papers written on the topic of wellness and health promotion for older adults in 2023–2024, it would likely result in a different set of research topics. Such results would make it possible for educators, researchers, and providers to anticipate and be prepared for those changes. Priorities and needs change as new research results are discovered, new types of training are accessed, and new efforts are made to address problems identified in past reports. The goal is that this Special Issue will inspire new and experienced academic professionals, healthcare providers, and public policy administrators to address and resolve current barriers or misunderstandings as they adopt new interests and priorities. After these issues are addressed, new action priorities will be adopted as more problems are uncovered and come to light. With global participation, new solutions will be found and applied to these new issues, exemplifying the continuous quality improvement cycle of education, medicine, and science. With the participation of all, the world will embrace the concepts of an age-friendly environment, becoming a safer place for all older adults. 

## Figures and Tables

**Table 1:** The 4Ms topics addressed in this Special Issue.

4Ms	Current Healthcare Concerns of Older Adults
What matters	Knowing What Matters to an Older Adult Freedom from food insecurity [10] Access to emergency services [11] Maintaining social interactions [12,13] Acknowledgement of their self-efficacy [14,15] Greater desire for autonomy than for safety [12] Access to a capable and sympathetic workforce [11,16,17,18] Maintaining physical strength and remaining resilient [19,20] Ability to use telehealth to access advice and assistance [21,22,23] Receiving respect for cultural beliefs and the value of lived experiences [16,19] Provider acceptance of wishes stated in advance directive, including end-of-life wishes [24,25,26]
Medications	Receiving Age-Friendly Management of Medications Allowing the use of traditional medicines [16] Attention to polypharmacy, deprescribing, and medication reconciliation [6,17,22,27] The use of screening for diabetes, hypertension, osteoporosis, and other common geriatric conditions [20,22]
Mobility	Being Offered Age-Friendly Mobility Plans Exercise and coordination plans [13,17,20] Falls screening, prediction, and prevention [27,28,29]
Mentation	Ensuring Age-Friendly Management of Mental Health Issues Screening for and discussing cognitive impairment [18,30,31] Encouraging social support and social engagement [20,32,33] Acknowledging and addressing loneliness, anxiety, and stress [14,19,24] Recognizing differences between dementia, depression, and delirium [10,17,27,33] Promoting communication between providers, older adults, and their caregivers [18] Training community health workers to enhance well-being and care delivery [34] Providing medical insurance [35]

## Data Availability

The conclusions discussed in this Editorial result from a distillation of the information provided in the papers published within this Special Issue.
